# Adherence to the acceleration of total postoperative recovery protocol and perioperative complications in cancer patients*

**DOI:** 10.1590/1980-220X-REEUSP-2025-0022en

**Published:** 2025-11-17

**Authors:** Silvia Paulino Ribeiro Albanese, Caroline Tolentino Sanches, Karine Silva de Oliveira, Maria Laura Albanese, Marcos Toshiyuki Tanita, Cíntia Magalhães Carvalho Grion

**Affiliations:** 1 Universidade Estadual de Londrina, Londrina, PR, Brazil.; 2 Pontifícia Universidade Católica, Londrina, PR, Brazil.

**Keywords:** Postoperative Complications, Hospital Mortality, Surgical Oncology, Intensive Care Units, Elective Surgical Procedures

## Abstract

**Objective:**

To describe postoperative complications in patients undergoing oncological surgeries, and to analyze adherence to the recommendations of the project Acceleration of Total Postoperative Recovery (*ACERTO*) in these patients and the risk factors for death.

**Method:**

Retrospective longitudinal study. Sample of 229 patients in the immediate postoperative period admitted to the intensive care unit from July to December 2021.

**Results:**

The frequency of complications was 68.5%. There was adherence to the recommendation of fluid resuscitation ≤ 30 mL/kg in 56.6% intraoperatively and greater adherence in the postoperative period (90.4%) and in the prescription of nausea and vomiting prophylaxis in the intraoperative (93%) and postoperative (100%) periods. An association was observed between adherence to recommendations and a reduction in complications. The independent risk factors for death were age (p = 0.031) and the score *Sequential Organ Failure Assessment* (SOFA) (p = 0.004).

**Conclusion:**

A high frequency of complications was observed in the postoperative period and a mortality rate of 11.8%. Adherence to the protocol ACERTO was associated with a reduction in postoperative complications in cancer patients. Age and SOFA score were independent risk factors for death.

## INTRODUCTION

Highly complex surgical interventions commonly require intensive care in the postoperative period, with recurrent admission to Intensive Care Units (ICU). This scenario is especially observed after cardiac, neurological procedures and extensive abdominal surgeries^(1)^. Oncological surgeries, in particular, stand out as procedures that require a specialized approach^(2)^.

Patients undergoing oncological surgeries face a series of specific risks that can significantly influence both the outcome of the procedure and postoperative recovery. The presence of metastases, indicative of the spread of cancer beyond the primary site, is an element that can influence surgical approaches and is associated with an increased risk of complications^(3)^.

Since the 1990s, the study and implementation of measures aimed at optimizing recovery in elective surgeries has been an area of interest. In Brazil, the project Acceleration of Total Postoperative Recovery (*ACERTO*) encompasses some of these protocols that are fundamental for the early identification of complications, evaluation of the effectiveness of the intervention, and optimization of the patients’ quality of life^(4)^. Furthermore, these practices contribute to uniformity in perioperative care, playing a significant role in optimizing clinical outcomes and improving the cancer patients’ overall condition. Its fundamental components include the provision of information and pre-habilitation, the reduction of the fasting period, and the early reintroduction of food, the implementation of appropriate prophylaxis and the correct management of symptoms, fluid resuscitation ≤ 30 mL/kg, and the non-preparation of the colon, as well as the adoption of minimally invasive procedures and the promotion of early mobilization. The pioneering application of the Project *ACERTO* resulted in a reduction in hospital stays, in the use of blood products, and a decrease in cases of surgical site infection, operative complications, and deaths^(5)^.

A thorough understanding of the risk factors for complications is essential for a more precise and effective approach to the postoperative management of patients undergoing oncological surgeries, aiming at the continuous improvement of clinical results and the promotion of quality of life. The objective of this study was to describe postoperative complications in patients undergoing oncological surgeries, and to analyze adherence to the recommendations of the project *ACERTO* in these patients and the risk factors for death.

## METHOD

### Design of Study

Retrospective cohort study.

### Local

Intensive care unit (ICU) at the Londrina Cancer Hospital, located in the northern region of Paraná. During the study period, the project *ACERTO* was not formally implemented as a protocol of the research institution. This project recommendations were used as a reference for the evaluation of clinical practice. Regarding infection prevention, the research institution has a formally implemented institutional protocol, covering several preventive measures aligned with the recommendations of the project *ACERTO*, such as restricting the use of drains and probes, early mobilization, early nutritional support in the postoperative period, and conduction of audits to evaluate results. The hospital infection control service team conducts daily audits, monitoring insertion and maintenance of invasive devices, dressings, signs of respiratory infection, and adherence to hand hygiene.

### Population and Selection Criteria

Adult patients aged 18 years or older, undergoing oncological surgical procedures and admitted to the ICU in the immediate postoperative period. Patients who underwent palliative surgery, as well as those who stayed in the ICU for less than 24 hours, were excluded from this study. Additionally, participants with missing data in their medical records that prevented the calculation of scores *Simplified Acute Physiology Score* 3 (SAPS 3) and *Sequential Organ Failure Assessment* (SOFA) were excluded, as well as those with lack of information on the occurrence or not of complications in the perioperative period.

### Sample Definition

Convenience sample of patients admitted to the study site between July and December 2021.

### Data Collection

The information collected covered the pre-, intra- and post-operative periods, extending up to the hospital outcome. The categorization of surgeries was based on the Table of the Management System for Procedures, Medications, Orthoses, Prostheses and Special Materials (*SIGTAP*) of the Brazilian Public Health System (*SUS*), a tool that allows access to the *SUS* procedure table.

Regarding sociodemographic, clinical, and epidemiological variables, data such as age, gender, pre-existing conditions (smoking, alcoholism, illicit drug use, cancer treatment and comorbidities) according to the *Charlson Comorbidity Index* ^(6)^ list were analyzed.

Preoperatively, variables included fasting time, length of preoperative hospital stay, need for blood transfusion, and colon preparation. Oncological treatments were considered to be chemotherapy and radiotherapy performed at any time before surgery for the treatment of the primary tumor.

During the surgical procedure, the classification of contamination potential, use of mechanical ventilation (MV), specific surgical procedure, type of anesthesia, administration of medications, volume replacement and use of vasoactive drugs were analyzed.

In the postoperative period, oxygen requirements, mechanical ventilation data, use of vasoactive agents, fluid resuscitation, fluid balance, need for hemodialysis, occurrence of bleeding, as well as the administration of analgesia and antiemetics were monitored.

Postoperative variables were recorded at five different times: immediate postoperative period (IPO), 1st postoperative period (1st PO), 2nd postoperative period (2nd PO), 3rd postoperative period (3rd PO), and last day of ICU stay. In addition to the variables previously mentioned, pertinent information was investigated, such as length of hospital and ICU stay, the occurrence of postoperative complications (infectious, cardiovascular, respiratory, gastrointestinal, renal, neurological, coagulation and electrolyte disorders), start of refeeding, reoperation, and clinical outcome. The clinical outcome was defined as the vital status at hospital discharge, with the groups divided into survivors (discharge) and non-survivors (death).

The SAPS 3 score assesses the severity of a patient’s condition at ICU admission to predict in-hospital mortality. SOFA score assesses the degree of organ dysfunction in six major organ systems (neurological, respiratory, cardiovascular, renal, hepatic, and hematological). These scores were the variables chosen because they are routinely used in clinical practice today. The SAPS 3 scores were assessed within the first hour of ICU admission, while the SOFA was assessed at the following time points: immediate postoperative period (IPO), 1st postoperative period (1st PO), 2nd postoperative period (2nd PO), 3rd postoperative period (3rd PO), and last day of ICU admission.

### Data Analysis and Treatment

The normality of the distribution of variables was verified using the Shapiro-Wilk test. The results of continuous variables were presented as mean, standard deviation (SD) or median, and interquartile range (IQR), depending on the data distribution. Student’s t-test was used to compare the means of continuous variables with normal distribution and homogeneity of variances. For data with non-normal distribution and/or heterogeneity of variances, the non-parametric test (Mann-Whitney U test) was applied.

Categorical variables were analyzed using the chi-square test and presented as absolute and relative frequency. The significance level adopted was 5%. The assessment of the association between potential risk factors (independent variables) and the dependent variable (hospital outcome) was conducted by presenting unadjusted odds ratios (OR) and 95% confidence intervals (95% CI). These data were obtained using the logistic regression model in Enter mode, which is characterized by bivariate analysis. Subsequently, the logistic regression model was adopted in the multivariate analysis. For the logistic regression analysis, the dependent variable was the non-survival outcome, the independent variables were age, sex, comorbidities, complications, SAPS 3, SOFA in the IPO, and type of surgery.

The sensitivity and specificity analysis of the SOFA score for outcome discrimination was performed using the ROC curve. Results are presented as area under the curve (AUC) and 95% confidence interval (95% CI). To test the statistically relevant differences between the curves, the differences in their areas were calculated two by two using the DeLong method. The entire analytical process was conducted using the software MedCalc® Statistical, version 22.018^(7)^.

### Ethical Aspects

This study was submitted to and approved by the UEL Human Research Ethics Committee (*CEP-UEL*) with opinion no. 3,900,546; CAAE no. 28310420.6.0000.5231. This research was exempted from the Free and Informed Consent Form due to the design and objectives of the study.

## RESULTS

To assess risk factors related to complications in patients undergoing postoperative oncological surgery, a sample consisting of patients consecutively admitted to the study site between July and December 2021 was selected. When applying the inclusion criteria, the sample resulted in 299 patients and, of these, 70 cases were excluded by the study criteria, leaving 229 cases for analysis. Exclusion occurred in situations where patients remained in the ICU for less than 24 hours, underwent palliative surgeries, or had missing data in their medical records.

The median age was higher among non-surviving patients (70 years ITQ 62–76) when compared to survivors (62 years ITQ 53–71; p = 0.025). A higher frequency of males was observed (117; 51.0%) and 198 (86.4%) of the cases presented comorbidities, with systemic arterial hypertension standing out (31; 57.2%). Regarding habits, the presence of smoking in 91 (39.7%) and alcoholism in 27 (11.8%) stands out. It was noted that 59 (25.8%) patients underwent some oncological treatment (radiotherapy or chemotherapy) prior to surgery. The predominant diagnosis associated with the surgical procedure was malignant neoplasm in the brain, totaling 38 (16.6%). Next, malignant neoplasm was observed in the rectosigmoid junction, rectum, anus and anal canal, with 24 (10.5%), and in malignant neoplasm in the trachea, bronchi and lungs, with 18 (7.9%). The most frequent surgery was neurosurgery (61; 26.6%) (Table 1).

**Table 1:** Characterization of patients undergoing surgical procedures admitted to the intensive care unit – Londrina, PR, Brazil, 2022.

Variables	Frequency	%
Male sex	117	51.0
Presence of Comorbidities		
SAH	31	57.2
DM	64	27.9
Heart disease	44	19.2
Hypothyroidism	21	9.2
CKD	19	8.3
COPD	16	7.0
PVD	2	0.9
Stroke	2	0.9
Liver cirrhosis	2	0.9
HIV/AIDS	1	0.4
Oncological Treatment at any time before surgery	59	25.8
Habits		
Smoking	91	39.7
Alcoholism	27	11.7
Surgery priority		
Elective	179	78.2
Urgent	50	21.8
Contamination potential		
Clean	128	55.9
Potentially Contaminated	77	33.6
Contaminated	23	10.0
Infected	01	0.4
Type of anesthesia		
General Anesthesia	118	51.5
Combined Anesthesia	100	43.7
Neuraxial anesthesia	11	4.8
Surgical procedure Sigtap subgroup		
Neurosurgery	61	26.6
Digestive System	38	16.6
Coloproctology	34	14.8
Thoracic Surgery	24	10.5
Urology	23	10.0
Head and Neck	18	7.9
Gynecology	10	4.4
Bones and Soft Tissues	8	3.5
Mastology	4	1.7
Other surgeries	4	1.7
Oral and maxillofacial	3	1.3
Skin and Plastic Surgery	1	0.4
Other surgeries in oncology	1	0.4

Legend: SAH = Systemic Arterial Hypertension; DM = Diabetes Mellitus; COPD = Chronic Obstructive Pulmonary Disease; CKD = Chronic Kidney Disease; PVD = Peripheral Vascular Disease; HIV = Human Immunodeficiency Virus, Sigtap = (Management System for the Table of Procedures, Medications, Orthoses/Prostheses and Special Materials of the SUS).

**Figure 1:**
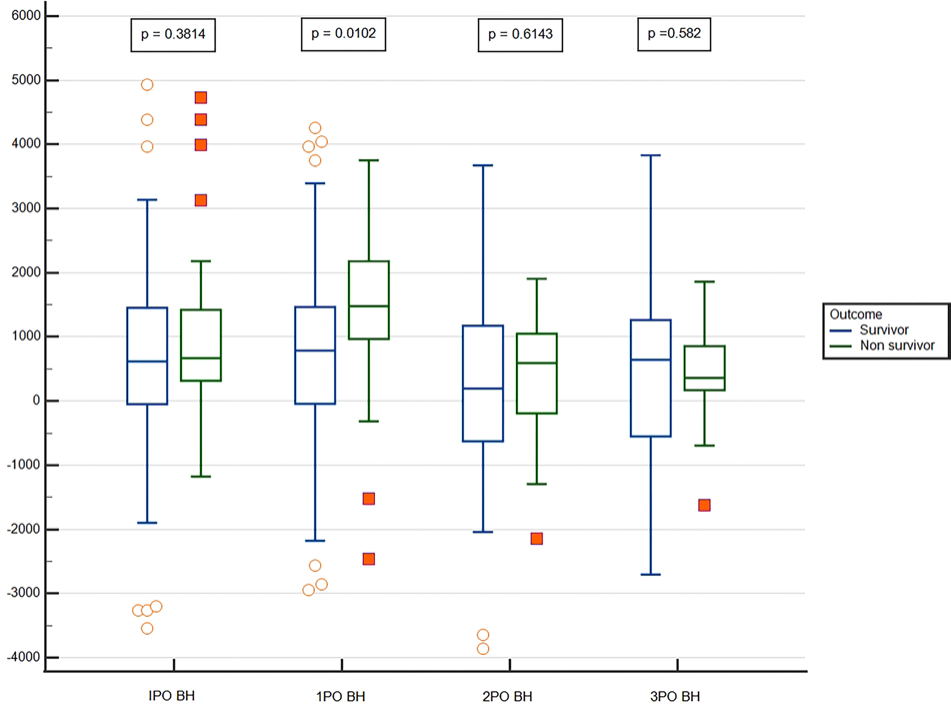
Comparison of fluid balance in patients during the postoperative period in the intensive care unit.

Legend: IPO = immediate postoperative period; 1PO = 1st postoperative period; 2PO = 2nd postoperative period; 3PO = 3rd postoperative period; FB = fluid balance.

Patients who had postoperative complications were exposed to a longer surgical time (205 min), while in patients who did not have complications this time was shorter (147 min) (p < 0.001). In the postoperative period, the fluid balance on the study days was: IPO: median = 619.4 mL (ITQ: -6.975–1,451.400); 1^st^ PO: median = 925 mL (ITQ: 0.425–1,745.200); 2^nd^ PO: median = 216 mL (ITQ: -469.000–1,153.250); 3^rd^ PO: 528 mL (ITQ: -444.600–1,193.750). The positive water balance in 1st PO was higher among non-survivors, 1,478.4 ml (ITQ: 969–218), compared to survivors, 786.2 ml (ITQ: -42–1,466; p = 0.010) (Figure 1).

The total frequency of postoperative complications was 157 (68.5%), with higher occurrences of infectious, respiratory, coagulation-related, and renal complications. Most of the complications analyzed in the postoperative period were associated with increased mortality, with the exception of gastrointestinal complications (Table 2). The mortality observed in this sample was 27 patients (11.8%).

**Table 2:** Bivariate analysis of postoperative complications as a risk factor for death – Londrina, PR, Brazil, 2022.

Complications	OR	CI 95%	p
Infectious	13.376	4.8124–37.1787	<0.001
Respiratory	10.773	4.4536–26.0631	<0.001
Coagulation	8.000	1.0788–59.3265	0.041
Renal	6.018	2.5549–14.1776	<0.001
Surgical	4.935	2.1381–11.3946	0.002
Neurological	4.582	1.7476–12.0138	0.002
Electrolytic	3.831	1.4872–9.8712	0.005
Cardiovascular	2.702	1.1774–6.2033	0.019
Gastric	1.987	0.8838–4.4680	0.096

Among the 72 cases with infectious complications, the most common were: pneumonia (34; 47.2%), surgical site infection (14; 19.4%), bloodstream infection (12; 16.7%), urinary tract infection (10; 13.9%), and undetermined origin (2; 2.8%). Of the patients with infectious complications, 14 (19.4%) presented with sepsis and 30 (41.6%) with septic shock. Among the 229 patients in the study, the proportion of patients who remained on MV for three or more days was 20 (8.7%). Among the 34 patients who developed pneumonia as an infectious complication, 12 (35.3%) remained on MV until the third day of follow-up. Of the 50 cases that presented surgical complications, 34 (68.0%) required intraoperative blood transfusion, 13 (26.0%) presented fistula, 16 (32.0%) had postoperative bleeding, and 7 (14.0%) had anastomotic rupture. The variable length of stay in the ICU was 44h19min among survivors and 96h04min among non-survivors. Readmissions to the ICU occurred in 16 (6.9%) cases, and reoperations in 17 (7.4%). SAPS 3 score did not differ when comparing non-survivors (median = 56; ITQ: 44–70) and survivors (median = 54; ITQ: 45–60; p = 0.129).

Adherence to the recommendations of the project *ACERTO* was observed in relation to early refeeding, volume replacement and prophylaxis of nausea and vomiting and their association with the occurrence of complications (Table 3). There was adherence to the recommendation of fluid resuscitation ≤ 30 mL/kg in 111 patients (56.6%) intraoperatively and 206 (90.4%) patients in the postoperative period, and prescription of nausea and vomiting prophylaxis in 213 patients (93%) intraoperatively and 229 (100%) patients postoperatively. There was lower adherence to the recommendation to shorten fasting (73; 32.0%). An association was observed between adherence to recommendations and a reduction in the frequency of complications (Table 3).

**Table 3:** Bivariate analysis of risk factors for postoperative complications in patients admitted to the intensive care unit considering the variables of the project Acceleration of Total Postoperative Recovery – Londrina, PR, Brazil, 2022.

Variables	Total	No complications	With complications	p
Postoperative refeeding				
Immediate up to 6 hours	73 (32.0%)	35 (47.9%)	38 (52.1%)	0.001
6 to 12 hours	37 (16.2%)	12 (32.4%)	25 (67.6%)	
12 to 24 hours	58 (25.4%)	16 (27.6%)	42 (72.4%)	
24 to 48 hours	38 (16.7%)	6 (15.8%)	32 (84.2%)	
Over 48 hours	21 (9.2%)	2 (9.5%)	19 (90.5%)	
Did not occur	1 (0.4%)	(–)	1 (100.0%)	
Intraoperative fluid resuscitation				
≤ 30 mL/kg	111(56.6%)	41 (36.9%)	70 (63.1%)	0.005
> 30 mL/kg	85 (43.4%)	16 (18.8%)	69 (81.2%)	
Fluid resuscitation in the immediate postoperative period				
≤ 30 mL/kg	206 (90.4%)	69 (33.5%)	137 (66.5%)	0.057
> 30 mL/kg	22 (9.6%)	3 (13.6%)	3 (13.6%)	
Fluid resuscitation				
No	34 (14.8%)	16 (47.1%)	18 (52.9%)	0.033
Yes	195 (85.2%)	56 (28.7%)	139 (71.3%)	
Positive balance in the immediate postoperative water balance				
No	201 (87.8%)	69 (34.3%)	132 (65.7%)	0.011
Yes	28 (12.2%)	3 (10.7%)	25 (89.3%)	
Prophylaxis of intraoperative nausea and vomiting				
No	16 (7.0%)	2 (12.5%)	14 (87.5%)	0.091
Yes	213 (93.0%)	70 (32.9%)	143 (67.1%)	
Prophylaxis of nausea and vomiting in the postoperative period				
No	(–)	(–)	(–)	<0.001
Yes	229 (100.0%)	72 (31.4%)	157 (68.6%)	

In multivariate analysis, the independent risk factors for death were: age (OR = 1.0388, 95% CI: 1.0034–1.0754; p = 0.031) and SOFA in the immediate postoperative period (OR = 1.2136, 95% CI: 1.0620–1.3867; p = 0.004) (Table 4).

**Table 4:** Bivariate and multivariate analysis for the death outcome – Londrina, PR, Brazil, 2022.

	Bivariate			Multivariate		
Variables	OR	CI 95%	p	OR	CI 95%	p
Age	1.037	1.0049–1.0710	0.023	1.038	1.0034–1.0754	0.031
Sex	1.035	0.4634–2.3123	0.933			
Comorbidities	4.534	0.5928–34.6894	0.145			
Complications	30.55	1.8366–508.3583	0.017			
SAPS 3	1.035	1.0058–1.0668	0.019			
SOFA at IPO	1.217	1.0687–1.3876	0.003	1.213	1.0620–1.3867	0.004
Surgery	1.604	0.6566–3.9186	0.299			

**Figure 2:**
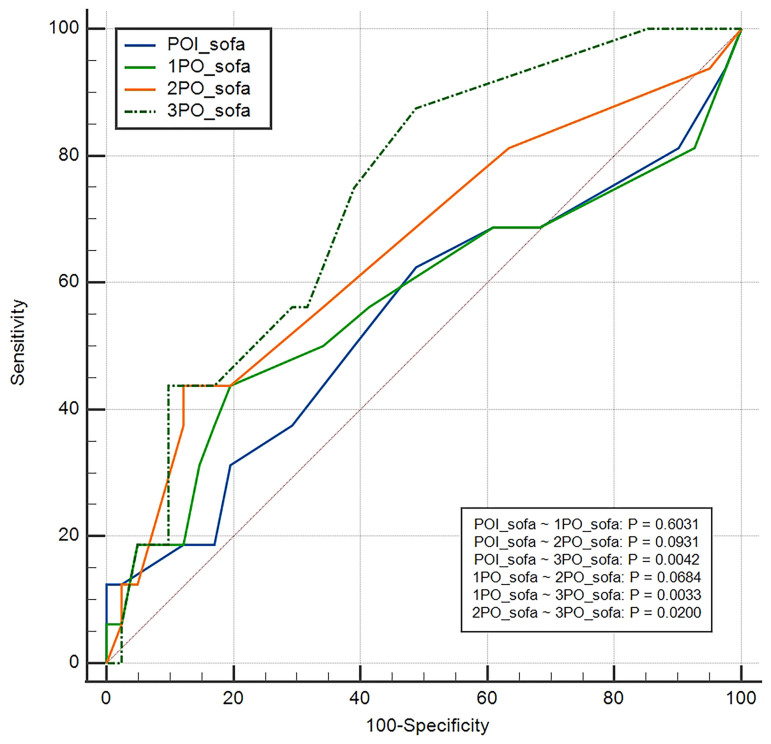
ROC curve to assess the effectiveness of the SOFA score as a predictor of postoperative mortality during ICU stay.

Legend: SOFA = *Sequential Organ Failure Assessment*; ICU = Intensive Care Unit; IPO = immediate postoperative period; 1PO = 1st postoperative period; 2PO = 2nd postoperative period; 3PO = 3rd postoperative period.

ROC curve analysis showed an AUC for SOFA in the immediate postoperative period of 0.625, in the 1st PO of 0.629, in the 2nd PO of 0.687, and in the 3rd PO of 0.721. SOFA scores on the 2nd PO (0.013) and 3rd PO (p = 0.002) showed greater discriminatory power to predict hospital mortality when compared to the others (Figure 2).

## DISCUSSION

In this study, adherence to the recommendations of the protocol *ACERTO* was associated with a reduction in postoperative complications in cancer patients admitted to the Intensive Care Unit. A high frequency of complications was observed, which were associated with a higher risk of death.

Study by Silva Jr et al.^(8)^ observed that most patients had an average age of 62 years, and most were male, as were the results found in the present study. In the sample analyzed, 86.5% of patients presented comorbidities. Other authors also show that advanced age represents a predictive factor for death, when associated with multiple comorbidities, especially regarding diabetes mellitus, arterial hypertension and polypharmacy, which contribute to the development of acute kidney injury and, consequently, a greater probability of death among intensive care patients^(9,10)^.

A high proportion of adherence to the ACERTO project recommendations for volume replacement ≤ 30 mL/kg was observed in the initial phases of hemodynamic resuscitation and prophylaxis of nausea and vomiting. Although the study site does not have a formal institutional protocol, these recommended practices are incorporated into the clinical routine. In a recent publication, a division of hemodynamic resuscitation into 4 phases was suggested, with fluid resuscitation recommended in the two initial phases with the aim of rapidly expanding intravascular volume to improve tissue perfusion^(11)^. This initial expansion may result in a positive water balance in these early stages. In the subsequent stabilization and de-escalation phases, the goal is a zero or even negative fluid balance, with the aim of preventing the adverse effects of volume overload. In the patients in the present study, we can observe a positive fluid balance in the first postoperative days consistent with this recommendation; however, a zero or negative value would be expected in the 3^rd^ PO. There were no patients prescribed fluid resuscitation among the patients in this study on the day of 3^rd^ PO; therefore, what would explain this positive water balance would be other fluids used in the treatment of critically ill patients, such as fluids for diluting medications, among others. Souza et. al. demonstrated that fluids that are not used for hemodynamic resuscitation can be the main contributors to a positive fluid balance during hemodynamic resuscitation^(12)^.

Surgical patients who are candidates for digestive procedures, especially in oncology, have a high prevalence of isolated malnutrition or malnutrition associated with sarcopenia^(13)^. In the present study, lower adherence to early refeeding was observed in the first 6 hours. A dose-response effect was also observed, that is, the longer the fasting in the postoperative period, the greater the frequency of complications observed. The prolonged pre- and post-operative fasting traditionally imposed by surgery can worsen the organic response and nutritional status, predisposing the patient to a greater response to trauma and impairment of the immune response. The protocol *ACERTO* recommends, with a strong degree of recommendation and a high level of evidence, that oral or enteral feeding after elective abdominal surgery should be early (within 24 hours postoperatively), as long as the patient is hemodynamically stable. In operations such as video cholecystectomy, hernioplasty, and ano-orificial surgeries, immediate initiation of diet and oral hydration is recommended, without the need for intravenous hydration^(14)^.

In the present study, crystalloid volume replacement ≤ 30 mL/kg in the early phases of hemodynamic resuscitation was associated with a lower occurrence of complications, corroborating the findings by other authors^(12)^. Crystalloid overload can cause widespread tissue edema, leading to several clinical consequences, such as impaired cardiopulmonary function, decreased blood oxygenation, and negative effects throughout the body. In the digestive tract, this results in splanchnic edema, increased intra-abdominal pressure, decreased mesenteric flow, maintenance of paralytic ileus, increased mucosal permeability, and impaired healing process^(15)^.

Another finding of the present study was the high adherence to prophylaxis recommendations for nausea and vomiting in the intraoperative and immediate postoperative period. A higher incidence of complications was observed among cases that did not receive prophylaxis. A study that analyzed the risk factors for postoperative nausea and vomiting (PONV) found that the incidence of PONV is high, reaching 37.5% after general anesthesia^(16)^. However, this incidence can reach 70% in high-risk patients^(17)^. It is important to note that the occurrence of nausea and vomiting delays the patient’s refeeding, undermines their confidence and increases the need for intravenous fluids and the risk of surgical wound dehiscence, which can lead to delayed hospital discharge^(15)^.

A mortality rate of 11.8% was identified during the hospitalization period, which is similar to the study carried out by Silva Jr et al.^(8)^, demonstrating an ICU mortality rate of 4.9%, hospital mortality rates of 8.9%, and 28-day follow-up death of 9.6%.

Postoperative complications associated with higher risks of death were infectious, respiratory, coagulation-related and renal. Among postoperative complications, the renal ones are responsible for a considerable proportion of morbidity and mortality related to surgeries^(18,19)^. A prospective, multicenter cohort study found the total incidence of postoperative complications, with higher occurrences of cardiovascular, renal, respiratory and neurological complications, respectively^(8)^.

Multivariate analysis identified independent risk factors for death, such as age and SOFA score. The importance of implementing mortality reduction strategies is highlighted, through the continuous evaluation of these scores by the ICU care and management staff in time to treat or reduce organic dysfunctions. In a prospective study of 100 ICU patients with sepsis and evidence of organ dysfunction, higher baseline SAPS 2 and SOFA scores were associated with worse outcomes^(20)^.

As a limitation of the study, it should be highlighted that it is a single-center and retrospective study, which may be restrictive and result in incomplete data on variables of interest such as those regarding *ACERTO* recommendations. The fact that it is conducted in a single center limits the number of participants and reduces the external validity of the results. Specifically regarding the analysis of fluid management, it was not possible to include data from intraoperative WB because records of fluid prescriptions were found during this period, but there was no data on losses, hindering this calculation. Furthermore, the limited sample size may have made it difficult to identify relevant risk factors for death, even if it was tested by the best-performing statistical model. The strength of this research lies in the fact that there is little Latin American data, especially in the oncology field.

The implication of these findings for clinical practice is emphasized, demonstrating the importance of implementing the project *ACERTO* in the hospital where the study was conducted, with the aim of reducing morbidity and mortality.

## CONCLUSION

This study identified a high frequency of complications in the postoperative period and a hospital mortality rate similar to that of other authors. Complications associated with higher risks of death were infectious, respiratory, renal, and coagulation-related. Adherence to the recommendations of the protocol *ACERTO* was associated with a reduction in postoperative complications in cancer patients. Age and SOFA score were observed as independent risk factors for death.

## DATA AVAILABILITY

The data for this research were deposited at the link: https://doi.org/10.48331/SCIELODATA.D4NF6L">https://doi.org/10.48331/SCIELODATA.D4NF6L.
