# Supplementary material for: Adherence to the acceleration of total postoperative recovery protocol and perioperative complications in cancer patients
Source: Rev Esc Enferm USP. 2025 Nov 17;59:e20250022. doi: 10.1590/1980-220X-REEUSP-2025-0022en (PMC12662594; doi:10.1590/1980-220X-REEUSP-2025-0022en)
Supplement: Translation

# Translation: Adesão ao protocolo aceleração da recuperação total pós-operatória e complicações perioperatórias em pacientes oncológicos*

**DOI:** 10.1590/1980-220X-REEUSP-2025-0022pt

**Authors:** Silvia Paulino Ribeiro Albanese, Caroline Tolentino Sanches, Karine Silva de Oliveira, Maria Laura Albanese, Marcos Toshiyuki Tanita, Cíntia Magalhães Carvalho Grion

**Affiliations:** 1 Universidade Estadual de Londrina, Londrina, PR, Brasil.; 2 Pontifícia Universidade Católica, Londrina, PR, Brasil.

**Keywords:** Complicações pós-operatórias, Mortalidade hospitalar, Oncologia Cirúrgica, Unidades de Terapia Intensiva, Procedimentos Cirúrgicos Eletivos

## Abstract

**Objetivo:**

Descrever as complicações pós-operatórias em pacientes submetidos a cirurgias oncológicas, analisar a adesão às recomendações do projeto Aceleração da Recuperação Total Pós-Operatória (ACERTO) nesses pacientes e os fatores de risco para morte.

**Método:**

Estudo longitudinal retrospectivo. Amostra de 229 pacientes em pós-operatório imediato admitidos na unidade de terapia intensiva no período de julho a dezembro de 2021.

**Resultados:**

A frequência de complicações foi de 68,5%. Houve adesão à recomendação de ressuscitação volêmica ≤ 30 mL/Kg em 56,6% no intraoperatório e maior adesão no pós-operatório (90,4%) e na prescrição de profilaxia de náuseas e vômitos nos períodos intraoperatório (93%) e pós-operatório (100%). Foi observada associação entre a adesão às recomendações e redução das complicações. Os fatores de risco independentes para o óbito foram idade (p = 0,031) e o escore *Sequential Organ Failure Assessment* (SOFA) (p = 0,004).

**Conclusão:**

Foi observada alta frequência de complicações no período pós-operatório e taxa de mortalidade de 11,8%. A adesão ao protocolo ACERTO foi associada à redução das complicações pós-operatórias em pacientes oncológicos. Idade e escore SOFA foram fatores de risco independentes para morte.

## INTRODUÇÃO

Intervenções cirúrgicas de alta complexidade comumente requerem atenção intensiva no pós-operatório, com internação em Unidades de Terapia Intensiva (UTI) de forma recorrente. Esse cenário é especialmente observado após procedimentos cardíacos, neurológicos e cirurgias abdominais extensas^(1)^. As cirurgias oncológicas, em especial, destacam-se como procedimentos que exigem uma abordagem especializada^(2)^.

Os pacientes submetidos a cirurgias oncológicas enfrentam uma série de riscos específicos que podem exercer uma influência significativa tanto no desfecho do procedimento quanto na recuperação pós-operatória. A presença de metástases, indicativo da disseminação do câncer para além do local primário, é um elemento que pode influenciar as abordagens cirúrgicas e está associada a um aumento do risco de complicações^(3)^.

Desde a década de 1990, o estudo e a implementação de medidas voltadas para otimizar a recuperação em cirurgias eletivas têm sido uma área de interesse. No Brasil, o projeto Aceleração da Recuperação Total Pós-Operatória (ACERTO) engloba alguns desses protocolos que são fundamentais para a identificação precoce de complicações, avaliação da eficácia da intervenção e otimização da qualidade de vida dos pacientes^(4)^. Além disso, essas práticas contribuem para a uniformidade nos cuidados perioperatórios, desempenhando um papel significativo na otimização dos resultados clínicos e no aprimoramento do estado geral do paciente oncológico. Seus componentes fundamentais englobam a disponibilização de informações e pré-habilitação, a redução do período de jejum e a reintrodução precoce da alimentação, a implementação de profilaxias apropriadas e o correto manejo de sintomas, a ressuscitação volêmica ≤ 30 mL/Kg e a não preparação do cólon, além da adoção de procedimentos minimamente invasivos e a promoção da mobilização precoce. A aplicação pioneira do Projeto ACERTO resultou na redução do tempo de internação hospitalar, do uso de hemoderivados e na diminuição de casos de infecção no local cirúrgico, complicações operatórias e óbitos^(5)^.

A compreensão aprofundada dos fatores de risco para complicações é essencial para uma abordagem mais precisa e eficaz no manejo pós-operatório dos pacientes submetidos a cirurgias oncológicas, visando a melhoria contínua dos resultados clínicos e a promoção da qualidade de vida. Esse estudo objetivou descrever as complicações pós-operatórias em pacientes submetidos a cirurgias oncológicas, analisar a adesão às recomendações do projeto ACERTO nesses pacientes e os fatores de risco para morte.

## MÉTODO

### Desenho do Estudo

Estudo de coorte retrospectivo.

### Local

Unidade de terapia intensiva do Hospital do Câncer de Londrina, situado na região norte do Paraná. No período do estudo não havia a implantação formal do projeto ACERTO como protocolo da instituição da pesquisa. As recomendações desse projeto foram utilizadas como referência para a avaliação da prática clínica. Em relação à prevenção de infecções, a instituição da pesquisa tem protocolo institucional formalmente implementado, abrangendo diversas medidas preventivas alinhadas com as recomendações do projeto ACERTO, como a restrição do uso de drenos e sondas, mobilização precoce, suporte nutricional precoce no pós-operatório e a realização de auditorias para avaliação de resultados. A equipe do serviço de controle de infecção hospitalar realiza auditorias diárias, monitorando inserção e manutenção de dispositivos invasivos, curativos, sinais de infecção respiratória e adesão à higiene das mãos.

### População e Critérios de Seleção

Pacientes adultos com 18 anos ou mais, submetidos a procedimentos cirúrgicos oncológicos e admitidos no pós-operatório imediato na UTI. Foram excluídos deste estudo os pacientes submetidos a cirurgias paliativas, assim como aqueles que permaneceram internados na UTI por menos de 24 horas. Também foram excluídos os participantes com dados faltantes no prontuário que impediam o cálculo dos escores *Simplified Acute Physiology Score* 3 (SAPS 3) e *Sequential Organ Failure Assessment* (SOFA), além da falta de informações sobre a ocorrência ou não de complicações no período perioperatório.

### Definição da Amostra

Amostra de conveniência dos pacientes admitidos no local de estudo no período compreendido entre julho e dezembro de 2021.

### Coleta de Dados

As informações captadas englobaram os períodos pré, intra e pós-operatório, estendendo-se até o desfecho hospitalar. A categorização das cirurgias foi feita com base na Tabela do Sistema de Gerenciamento de Procedimentos, Medicamentos, Órteses, Próteses e Materiais Especiais (SIGTAP) do Sistema Único de Saúde SUS, uma ferramenta que possibilita o acesso à tabela de procedimentos do SUS.

No que diz respeito às variáveis sociodemográficas, clínicas e epidemiológicas, foram analisados dados como idade, gênero, condições pré-existentes (tabagismo, alcoolismo, uso de drogas ilícitas, tratamento oncológico e comorbidades conforme lista de Charlson *Comorbidity Index* ^(6)^.

No pré-operatório, as variáveis incluíram tempo de jejum, tempo de internação pré-operatória, necessidade de transfusão sanguínea e realização de preparo de cólon. Foram considerados tratamentos oncológicos a quimioterapia e a radioterapia realizados em qualquer momento antes da cirurgia para o tratamento do tumor primário.

No procedimento cirúrgico, foram analisadas classificação do potencial de contaminação, uso de ventilação mecânica (VM), procedimento cirúrgico específico, tipo de anestesia, administração de medicações, reposição volêmica e utilização de drogas vasoativas.

No período pós-operatório foram monitoradas necessidade de oxigênio, dados da ventilação mecânica, uso de agentes vasoativos, reposição volêmica, balanço hídrico, necessidade de hemodiálise, ocorrência de sangramento, bem como a administração de analgesia e antieméticos.

As variáveis pós-operatórias foram registradas em cinco momentos distintos: pós-operatório imediato (POI), 1º pós-operatório (1º PO), 2º pós-operatório (2º PO), 3º pós- operatório (3º PO) e último dia de internação na UTI. Além das variáveis anteriormente mencionadas, foram investigadas informações pertinentes, como o tempo de permanência hospitalar e na UTI, a ocorrência de complicações pós-operatórias (infecciosas, cardiovasculares, respiratórias, gastrointestinais, renais, neurológicas, distúrbio de coagulação e eletrolíticas), início da realimentação, reoperação e desfecho clínico. O desfecho clínico foi definido como o estado vital na alta hospitalar, sendo os grupos divididos em sobreviventes (alta) e não sobreviventes (óbito).

O escore SAPS 3 avalia a gravidade da condição de um paciente na admissão em UTI para prever a mortalidade hospitalar. O escore SOFA avalia o grau de disfunção orgânica em seis sistemas orgânicos principais (neurológico, respiratório, cardiovascular, renal, hepático e hematológico). Esses escores foram as variáveis escolhidas por serem de uso rotineiro na prática clínica atualmente. A avaliação dos escores SAPS 3 foi realizada na primeira hora de internação na UTI, enquanto o SOFA foi avaliado nos seguintes momentos: pós-operatório imediato, 1º pós-operatório, 2º pós-operatório, 3º pós-operatório e último dia de internação na UTI.

### Análise e Tratamento dos Dados

A normalidade da distribuição das variáveis foi verificada por meio do teste de Shapiro-Wilk. Os resultados das variáveis contínuas foram apresentados como média, desvio padrão (DP) ou mediana e intervalo interquartílico (ITQ), dependendo da distribuição dos dados. O teste t de Student foi empregado para comparação das médias das variáveis contínuas com distribuição normal e homogeneidade de variâncias. Para dados com distribuição não normal e/ou heterogeneidade de variâncias, aplicou-se o teste não paramétrico (teste U de Mann-Whitney).

As variáveis categóricas foram analisadas utilizando o teste qui-quadrado, sendo apresentadas como frequência absoluta e relativa. O nível de significância adotado foi de 5%. A avaliação da associação entre potenciais fatores de risco (variáveis independentes) e a variável dependente (desfecho hospitalar) foi conduzida por meio da apresentação de odds ratio (OR) não-ajustado e intervalo de confiança de 95% (IC 95%). Esses dados foram obtidos utilizando-se o modelo de regressão logística em modo Enter, o qual é caracterizado pela análise bivariada. Posteriormente, adotou-se o modelo de regressão logística na análise multivariada. Para a análise de regressão logística, a variável dependente foi o desfecho não sobrevivente, as variáveis independentes foram idade, sexo, comorbidades, complicações, SAPS 3, SOFA no POI e tipo de cirurgia.

A análise de sensibilidade e especificidade do escore SOFA para a discriminação do desfecho foi realizada pela curva ROC. Os resultados são apresentados como área sob a curva (AUC) e intervalo de confiança de 95% (IC 95%). Para testar as diferenças estatisticamente relevantes entre as curvas, as diferenças das suas áreas foram calculadas duas a duas por meio do método de DeLong. Todo o processo analítico foi conduzido utilizando-se o programa MedCalc® Statistical Software version 22.018^(7)^.

### Aspectos Éticos

O presente estudo foi submetido e aprovado pelo Comitê de Ética em Pesquisa Envolvendo Seres Humanos da UEL (CEP-UEL) com o parecer nº 3.900.546; CAAE nº: 28310420.6.0000.5231. Esta pesquisa obteve dispensa do Termo de Consentimento Livre e Esclarecido justificada pelo delineamento e objetivos do estudo.

## RESULTADOS

Para a avaliação dos fatores de risco relacionados a complicações em pacientes submetidos ao pós-operatório de cirurgia oncológica, foi selecionada uma amostra composta por pacientes admitidos consecutivamente no local do estudo no período de julho a dezembro de 2021. Ao aplicar os critérios de inclusão, a amostra resultou em 299 pacientes e, dentre esses, 70 casos foram excluídos pelos critérios do estudo, restando 229 casos para análise. A exclusão ocorreu em situações em que os pacientes permaneceram na UTI por menos de 24 horas, foram submetidos a cirurgias paliativas ou apresentaram dados ausentes nos prontuários.

A mediana da idade foi maior entre os pacientes não sobreviventes (70 anos ITQ 62–76) quando comparada aos sobreviventes (62 anos ITQ 53–71; p = 0,025). Foi observada maior frequência do sexo masculino (117; 51,0%) e 198 (86,4%) dos casos apresentavam comorbidades, destacando-se hipertensão arterial sistêmica (31; 57,2%). Quanto aos hábitos, destaca- se a presença de tabagismo em 91 (39,7%) e etilismo em 27 (11,8%). Notou-se que 59 (25,8%) pacientes foram submetidos a algum tratamento oncológico (radioterapia ou quimioterapia) em momento prévio à cirurgia. O diagnóstico predominante associado ao procedimento cirúrgico foi neoplasia maligna no encéfalo, totalizando 38 (16,6%). Em seguida, observou-se a neoplasia maligna na junção retosigmoide, reto, ânus e canal anal, com 24 (10,5%), e a neoplasia maligna na traqueia, brônquios e pulmões, com 18 (7,9%). A cirurgia mais frequente foi neurocirurgia (61; 26,6%) (Tabela 1).

**Tabela 1:** Caracterização dos pacientes submetidos a procedimentos cirúrgicos admitidos na unidade de terapia intensiva – Londrina, PR, Brasil, 2022.

Variáveis	Frequência	%
Sexo Masculino	117	51,0
Presença de Comorbidades		
HAS	31	57,2
DM	64	27,9
Cardiopatia	44	19,2
Hipotireoidismo	21	9,2
DRC	19	8,3
DPOC	16	7,0
DVP	2	0,9
AVE	2	0,9
Cirrose hepática	2	0,9
HIV/Aids	1	0,4
Tratamento Oncológico em qualquer momento antes da cirurgia	59	25,8
Hábitos		
Tabagismo	91	39,7
Etilismo	27	11,7
Prioridade da cirurgia		
Eletiva	179	78,2
Urgência	50	21,8
Potencial de contaminação		
Limpa	128	55,9
Potencialmente Contaminada	77	33,6
Contaminada	23	10,0
Infectada	01	0,4
Tipo de anestesia		
Anestesia Geral	118	51,5
Anestesia Combinada	100	43,7
Anestesia Neuroaxial	11	4,8
Procedimento cirúrgico Subgrupo Sigtap		
Neurocirurgia	61	26,6
Aparelho Digestivo	38	16,6
Coloproctologia	34	14,8
Cirurgia Torácica	24	10,5
Urologia	23	10,0
Cabeça e Pescoço	18	7,9
Ginecologia	10	4,4
Ossos e Partes Moles	8	3,5
Mastologia	4	1,7
Outras cirurgias	4	1,7
Bucomaxilofacial	3	1,3
Pele e Cirurgia Plástica	1	0,4
Outras cirurgias em oncologia	1	0,4

Legenda: HAS = Hipertensão Arterial Sistêmica; DM = Diabetes Mellitus; DPOC = Doença Pulmonar Obstrutiva Crônica; DRC = Doença Renal Crônica; DVP = Doença Vascular Periférica; AVE = Acidente Vascular Encefálico; HIV = Vírus da Imunodeficiência Humana, Sigtap = (Sistema de Gerenciamento da Tabela de Procedimentos, Medicamentos, Órteses/Próteses e Materiais Especiais do SUS).

Os pacientes que tiveram complicações pós-operatórias foram expostos a um maior tempo cirúrgico (205 min), enquanto nos pacientes que não tiveram complicações esse tempo foi menor (147 min) (p < 0,001).

No período pós-operatório, o saldo do balanço hídrico nos dias do estudo foi: POI: mediana = 619,4 mL (ITQ: -6,975–1.451,400); 1o PO: mediana = 925 mL (ITQ: 0,425–1.745,200); 2o PO: mediana = 216 mL (ITQ: -469,000–1.153,250); 3o PO: 528 mL (ITQ: -444,600–1.193,750). O saldo positivo do balanço hídrico no 1o PO foi maior entre os não sobreviventes, 1.478,4 ml (ITQ: 969–218), comparado aos sobreviventes, 786,2 ml (ITQ: -42–1.466; p = 0,010) (Figura 1).

**Figura 1:** Comparação do saldo do balanço hídrico nos pacientes durante o pós-operatório na unidade de terapia intensiva.

Legenda: UTI = Unidade de Terapia Intensiva; IPO = pós-operatório imediato; 1PO = 1º pós-operatório; 2PO = 2º pós-operatório; 3PO = 3º pós-operatório; BH = balanço hídrico.

A frequência total de complicações pós-operatórias foi de 157 (68,5%), com maiores ocorrências de complicações infecciosas, respiratórias, relativas à coagulação e renais. A maior parte das complicações analisadas no pós-operatório esteve associada a aumento de mortalidade, com exceção das complicações gastrointestinais (Tabela 2). A mortalidade observada nessa amostra foi de 27 pacientes (11,8%).

**Tabela 2:** Análise bivariada das complicações pós-operatórias como fator de risco para o óbito – Londrina, PR, Brasil, 2022.

Complicações	OR	IC 95%	p
Infecciosas	13,376	4,8124–37,1787	<0,001
Respiratórias	10,773	4,4536–26,0631	<0,001
Coagulação	8,000	1,0788–59,3265	0,041
Renais	6,018	2,5549–14,1776	<0,001
Cirúrgicas	4,935	2,1381–11,3946	0,002
Neurológicas	4,582	1,7476–12,0138	0,002
Eletrolíticas	3,831	1,4872–9,8712	0,005
Cardiovasculares	2,702	1,1774–6,2033	0,019
Gástricas	1,987	0,8838–4,4680	0,096

Dentre os 72 casos com complicações infecciosas, as mais comuns foram: pneumonia (34; 47,2%), infecção de sítio cirúrgico (14; 19,4%), infecção de corrente sanguínea (12; 16,7%), infecção de trato urinário (10; 13,9%) e foco de origem indeterminada (2; 2,8%). Dos pacientes com complicações infecciosas, 14 (19,4%) apresentaram-se como sepse e 30 (41,6%) como choque séptico. Entre os 229 pacientes do estudo, a proporção de pacientes que permaneceram em VM por três ou mais dias foi 20 (8,7%). Entre os 34 pacientes que desenvolveram pneumonia como complicação infecciosa, 12 (35,3%) permaneciam em VM até o terceiro dia de acompanhamento. Dos 50 casos que apresentaram complicações cirúrgicas, 34 (68,0%) necessitaram de transfusão sanguínea no intraoperatório, 13 (26,0%) apresentaram fístula, 16 (32,0%) tiveram sangramento no pós-operatório, 7 (14,0%) ruptura de anastomose. A variável tempo de internação na UTI foi de 44h19min entre os sobreviventes e 96h04min entre os não sobreviventes. As readmissões na UTI ocorreram em 16 (6,9%) casos, e as reoperações em 17 (7,4%). O escore SAPS 3 não diferiu ao comparar não sobreviventes (mediana = 56; ITQ: 44–70) e sobreviventes (mediana = 54; ITQ: 45–60; p = 0,129).

Foi observada adesão às recomendações do projeto ACERTO em relação a realimentação precoce, reposição volêmica e profilaxia de náuseas e vômitos e sua associação com a ocorrência de complicações (Tabela 3). Houve adesão à recomendação de ressuscitação volêmica ≤ 30 mL/Kg no intraoperatório em 111 pacientes (56,6%) e pós-operatório em 206 pacientes (90,4%) e prescrição de profilaxia de náuseas e vômitos no intraoperatório em 213 pacientes (93,0%) e pós-operatório em 229 pacientes (100,0%). Houve menor adesão à recomendação de abreviação do jejum (73; 32,0%). Foi observada associação entre a adesão às recomendações e redução da frequência de complicações (Tabela 3).

**Tabela 3:** Análise bivariada dos fatores de risco para complicações pós-operatórias em pacientes admitidos na unidade de terapia intensiva considerando as variáveis do projeto Aceleração da Recuperação Total Pós-Operatória – Londrina, PR, Brasil, 2022.

Variáveis	Total	Sem complicações	Com complicações	p
Realimentação pós-operatória				
Imediato até 6 horas	73 (32,0%)	35 (47,9%)	38 (52,1%)	0,001
6 a 12 horas	37 (16,2%)	12 (32,4%)	25 (67,6%)	
12 a 24 horas	58 (25,4%)	16 (27,6%)	42 (72,4%)	
24 a 48 horas	38 (16,7%)	6 (15,8%)	32 (84,2%)	
Acima de 48 horas	21 (9,2%)	2 (9,5%)	19 (90,5%)	
Não ocorreu	1 (0,4%)	(–)	1 (100,0%)	
Reposição volêmica intraoperatória				
≤ 30 mL/Kg	111(56,6%)	41 (36,9%)	70 (63,1%)	0,005
> 30 mL/Kg	85 (43,4%)	16 (18,8%)	69 (81,2%)	
Reposição volêmica no pós-operatório imediato				
≤ 30 mL/Kg	206 (90,4%)	69 (33,5%)	137 (66,5%)	0,057
> 30 mL/Kg	22 (9,6%)	3 (13,6%)	3 (13,6%)	
Reposição volêmica				
Não	34 (14,8%)	16 (47,1%)	18 (52,9%)	0,033
Sim	195 (85,2%)	56 (28,7%)	139 (71,3%)	
Saldo positivo no Balanço hídrico do pós-operatório imediato				
Não	201 (87,8%)	69 (34,3%)	132 (65,7%)	0,011
Sim	28 (12,2%)	3 (10,7%)	25 (89,3%)	
Profilaxia de náuseas e vômitos intraoperatório				
Não	16 (7,0%)	2 (12,5%)	14 (87,5%)	0,091
Sim	213 (93,0%)	70 (32,9%)	143 (67,1%)	
Profilaxia de náuseas e vômitos no Pós-operatório				
Não	(–)	(–)	(–)	<0,001
Sim	229 (100,0%)	72 (31,4%)	157 (68,6%)	

Em análise multivariada os fatores de risco independentes para o óbito foram: idade (OR= 1,0388, IC 95%: 1,0034–1,0754; p = 0,031) e SOFA no pós-operatório imediato (OR = 1,2136, IC 95%: 1,0620–1,3867; p = 0,004) (Tabela 4).

**Tabela 4:** Análise bivariada e multivariada para o desfecho óbito – Londrina, PR, Brasil, 2022.

	Bivariado			Multivariado		
Variáveis	OR	IC 95%	p	OR	IC 95%	p
Idade	1,037	1,0049–1,0710	0,023	1,038	1,0034–1,0754	0,031
Sexo	1,035	0,4634–2,3123	0,933			
Comorbidades	4,534	0,5928–34,6894	0,145			
Complicações	30,55	1,8366–508,3583	0,017			
SAPS 3	1,035	1,0058–1,0668	0,019			
SOFA no POI	1,217	1,0687–1,3876	0,003	1,213	1,0620–1,3867	0,004
Cirurgia	1,604	0,6566–3,9186	0,299			

A análise da curva ROC mostrou AUC para o SOFA no pós-operatório imediato de 0,625, no 1º PO de 0,629, no 2º PO de 0,687 e no 3º PO de 0,721. Os escores SOFA no 2º PO (0,013) e no 3º PO (p = 0,002) apresentaram maior poder de discriminação para predizer mortalidade hospitalar quando comparados aos demais (Figura 2).

**Figura 2:** Curva ROC para avaliação da eficácia do escore SOFA como preditor de mortalidade no pós-operatório durante a internação na UTI.

Legenda: SOFA = *Sequential Organ Failure Assessment*; UTI = Unidade de Terapia Intensiva; IPO = pós-operatório imediato; 1PO = 1º pós-operatório; 2PO = 2º pós-operatório; 3PO = 3º pós-operatório.

## DISCUSSÃO

Neste estudo a adesão às recomendações do protocolo ACERTO associou-se à redução das complicações pós- operatórias nos pacientes oncológicos admitidos em Unidade de Terapia Intensiva. Foi observada alta frequência de complicações, as quais estiveram associadas a maior risco de óbito.

Estudo de Silva Jr et al.^(8)^ observou que a maioria dos pacientes tinha idade média de 62 anos, sendo a maior parte do sexo masculino, assim como os resultados encontrados no presente estudo. Na amostra analisada, 86,5% dos pacientes apresentaram comorbidades. Outros autores também mostram que a idade avançada representa um fator preditivo para morte, quando associada a múltiplas comorbidades, principalmente no que concerne a diabetes mellitus, hipertensão arterial e uso de polifarmácia, que contribuem para o desenvolvimento de lesão renal aguda e, consequentemente, maior probabilidade de óbito entre os pacientes de cuidados intensivos^(9,10)^.

Foi observada alta proporção de adesão às recomendações do projeto ACERTO para reposição volêmica ≤ 30 mL/Kg nas fases iniciais da ressuscitação hemodinâmica e profilaxia de náuseas e vômitos. Embora o local de estudo não possua um protocolo institucional formal, essas práticas recomendadas estão incorporadas na rotina clínica. Em recente publicação foi sugerida uma divisão da ressuscitação hemodinâmica em 4 fases, sendo que nas duas fases iniciais se recomenda reposição de fluidos com o objetivo de rápida expansão do volume intravascular para melhorar a perfusão tecidual^(11)^. Essa expansão inicial pode resultar em balanço hídrico positivo nessas fases iniciais. Nas fases seguintes de estabilização e de-escalonamento o objetivo é um balanço hídrico zero ou mesmo negativo, com o objetivo de prevenir os efeitos adversos da sobrecarga de volume. Nos pacientes do presente estudo, podemos notar um balanço hídrico positivo nos primeiros dias de pós-operatório compatível com essa recomendação, porém seria esperado um valor zero ou negativo no 3o PO. Não havia pacientes com prescrição de ressuscitação volêmica nos pacientes do presente estudo nesse dia de 3o PO, portanto o que explicaria esse balanço hídrico positivo seriam outros fluidos utilizados no tratamento do paciente grave, como fluidos para diluição de medicamentos, entre outros. Souza e colaboradores demonstraram que fluidos que não são utilizados para ressuscitação hemodinâmica podem ser os contribuintes principais para um balanço hídrico positivo durante a ressuscitação hemodinâmica^(12)^.

Pacientes cirúrgicos candidatos a procedimentos digestivos, principalmente em oncologia, apresentam alta prevalência de desnutrição isolada ou associada à sarcopenia^(13)^. No presente estudo, observou-se menor adesão à realimentação precoce nas primeiras 6 horas. Também foi observado um efeito de dose resposta, ou seja, quanto mais longo o jejum no período pós-operatório, maior a frequência de complicações observadas. O prolongado jejum pré e pós-operatório tradicionalmente imposto pela cirurgia pode agravar a resposta orgânica e o estado nutricional, predispondo o paciente a uma maior resposta ao trauma e prejuízo da resposta imunológica. O protocolo ACERTO recomenda, com forte grau de recomendação e alto nível de evidência, que a realimentação oral ou enteral após operação abdominal eletiva deve ser precoce (dentro de 24 horas de pós-operatório), desde que o paciente esteja hemodinamicamente estável. Em operações como videocolecistectomia, hernioplastias e cirurgias ano-orificiais, recomenda-se o início imediato de dieta e hidratação oral, sem necessidade de hidratação intravenosa^(14)^.

No presente estudo, a reposição volêmica de cristaloides ≤ 30 mL/Kg nas primeiras fases da ressuscitação hemodinâmica associou-se com menor ocorrência de complicações, corroborando achado de outros autores^(12)^. A sobrecarga de cristaloides pode causar edema generalizado de tecidos, levando a várias consequências clínicas, como a perturbação da função cardiopulmonar, diminuição da oxigenação sanguínea e efeitos negativos em todo o organismo. No trato digestivo, isso resulta em edema esplâncnico, aumento da pressão intra-abdominal, diminuição do fluxo mesentérico, manutenção do íleo paralítico, aumento da permeabilidade da mucosa e comprometimento do processo de cicatrização^(15)^.

Outro achado do presente estudo foi a alta adesão às recomendações de profilaxia para náuseas e vômitos no intraoperatório e no período pós-operatório imediato. Observou-se uma maior incidência de complicações entre os casos que não receberam a profilaxia. Um estudo que analisou os fatores de risco para náuseas e vômitos pós-operatórios (NVPO) constatou que a incidência de NVPO é alta, alcançando 37,5% após anestesia geral^(16)^. No entanto, essa incidência pode chegar a 70% em pacientes de alto risco^(17)^. Destaca-se que a ocorrência de náuseas e vômitos retarda a realimentação do paciente, abala sua confiança e aumenta a necessidade de líquidos intravenosos e risco de deiscência de ferida operatória, o que pode levar ao atraso da alta hospitalar^(15)^.

Foi identificada mortalidade de 11,8% durante o período de internação que se assemelha ao estudo realizado por Silva Jr et al.^(8)^, demonstrando taxa de mortalidade na UTI de 4,9%, taxas de mortalidade hospitalar de 8,9%, e aos 28 dias de seguimento de 9,6%.

As complicações pós-operatórias associadas a maiores riscos para óbito foram: infecciosas, respiratórias, relacionadas a coagulação e renais. Dentre as complicações pós-operatórias, as renais são responsáveis por proporção considerável de morbidade e mortalidade relacionadas às cirurgias^(18,19)^. Estudo de coorte prospectivo, multicêntrico, constatou a incidência total de complicações pós-operatórias, com maiores ocorrências de complicações cardiovasculares, renais, respiratórias e neurológicas respectivamente^(8)^.

A análise multivariada identificou fatores de risco independentes para a morte, como a idade e o escore SOFA. Destaca-se a importância da implementação de estratégias de redução da mortalidade, mediante a avaliação contínua destes escores pelo corpo assistencial e gerencial das UTIs a tempo de tratar ou reduzir disfunções orgânicas. Em um estudo prospectivo que analisou 100 pacientes com sepse internados em UTI com evidência de disfunção orgânica, pontuações iniciais mais altas do SAPS 2 e SOFA foram associadas a piores desfechos^(20)^.

Como limitação do estudo, destaca-se que se trata de um estudo de centro único e retrospectivo, o que pode ser restritivo e resultar em dados incompletos sobre variáveis de interesse como aquelas a respeito de recomendações do ACERTO. O fato de ser conduzido em um único centro limita o número de participantes e reduz a validade externa dos resultados. Especificamente sobre a análise do manejo de fluidos, não foi possível incluir dados do BH intraoperatório pois foram encontrados registros da prescrição de fluidos nesse período, mas não havia dados sobre as perdas, impossibilitando esse cálculo. Além disso, o tamanho amostral limitado pode ter dificultado a identificação de fatores de risco relevantes para óbito, mesmo que tenha sido testado pelo modelo estatístico com melhor desempenho. A força desta pesquisa reside no fato de que existem poucos dados latino-americanos, especialmente na área oncológica.

Ressalta-se a implicação desses achados para a prática clínica, demonstrando a importância de implementar o projeto ACERTO no hospital do local de estudo, com o objetivo de reduzir a morbidade e mortalidade.

## CONCLUSÃO

Neste estudo foi identificada alta frequência de complicações no período pós-operatório e taxa de mortalidade hospitalar semelhante a outros autores. As complicações associadas a maior risco para óbito foram infecciosas, respiratórias, renais e relacionadas a coagulação. A adesão às recomendações do protocolo ACERTO associou-se à redução das complicações pós-operatórias no paciente oncológico. Observaram-se como fatores de risco independentes para morte a idade e o escore SOFA.

## DISPONIBILIDADE DE DADOS

Os dados da presente pesquisa foram depositados no link: https://doi.org/10.48331/SCIELODATA.D4NF6L.

# Translation: Adhesión al protocolo aceleración de la recuperación total posoperatoria y complicaciones perioperatorias en pacientes oncológicos*

**DOI:** 10.1590/1980-220X-REEUSP-2025-0022es

**Authors:** Silvia Paulino Ribeiro Albanese, Caroline Tolentino Sanches, Karine Silva de Oliveira, Maria Laura Albanese, Marcos Toshiyuki Tanita, Cíntia Magalhães Carvalho Grion

**Affiliations:** 1 Universidade Estadual de Londrina, Londrina, PR, Brasil.; 2 Pontifícia Universidade Católica, Londrina, PR, Brasil.

**Keywords:** Complicaciones Posoperatorias, Mortalidad Hospitalaria, Oncología Quirúrgica, Unidades de Cuidados Intensivos, Procedimientos Quirúrgicos Electivos

## Abstract

**Objetivo:**

Describir las complicaciones postoperatorias en pacientes sometidos a cirugías oncológicas y analizar la adherencia a las recomendaciones del proyecto Aceleración de la recuperación postoperatoria total (*ACERTO*) en estos pacientes y los factores de riesgo de muerte.

**Método:**

Estudio longitudinal retrospectivo. Muestra de 229 pacientes en postoperatorio inmediato ingresados en la unidad de cuidados intensivos de julio a diciembre de 2021.

**Resultados:**

La frecuencia de complicaciones fue del 68,5%. Hubo adherencia a la recomendación de reanimación con volumen ≤ 30 mL/kg en el 56,6% intraoperatoriamente y mayor adherencia en el postoperatorio (90,4%) y en la prescripción de profilaxis de náuseas y vómitos en el intraoperatorio (93%) y postoperatorio (100%). Se observó una asociación entre la adherencia a las recomendaciones y una reducción de las complicaciones. Los factores de riesgo independientes de muerte fueron la edad (p = 0,031) y la puntuación *Sequential Organ Failure Assessment* (SOFA) (p = 0,004).

**Conclusión:**

Se observó una alta frecuencia de complicaciones en el postoperatorio y una tasa de mortalidad del 11,8%. La adherencia al protocolo *ACERTO* se asoció con una reducción de las complicaciones postoperatorias en pacientes con cáncer. La edad y la puntuación SOFA fueron factores de riesgo independientes de muerte.

## INTRODUCCIÓN

Las intervenciones quirúrgicas de alta complejidad suelen requerir cuidados intensivos en el posoperatorio, con ingresos recurrentes en unidades de terapia intensiva (UTI). Esta situación se observa especialmente después de procedimientos cardíacos, neurológicos y cirugías abdominales extensas^(1)^. Las cirugías oncológicas, en particular, se destacan como procedimientos que requieren un enfoque especializado^(2)^.

Los pacientes sometidos a cirugías oncológicas enfrentan una serie de riesgos específicos que pueden influir significativamente tanto en el resultado del procedimiento como en la recuperación posoperatoria. La presencia de metástasis, indicativa de la diseminación del cáncer más allá del sitio primario, es un elemento que puede influir en los enfoques quirúrgicos y se asocia con un mayor riesgo de complicaciones^(3)^.

Desde la década de 1990, el estudio y la implementación de medidas destinadas a optimizar la recuperación en cirugías electivas han sido un área de interés. En Brasil, el proyecto Aceleración de la Recuperación Total Postoperatoria (ACERTO) engloba algunos de estos protocolos que son fundamentales para la identificación temprana de complicaciones, la evaluación de la eficacia de la intervención y la optimización de la calidad de vida de los pacientes^(4)^. Además, estas prácticas contribuyen a la uniformidad en los cuidados perioperatorios, desempeñando un papel significativo en la optimización de los resultados clínicos y en la mejora del estado general del paciente oncológico. Sus componentes fundamentales abarcan la disponibilidad de información y la pre-habilitación, la reducción del período de ayuno y la reintroducción temprana de la alimentación, la implementación de profilaxis adecuadas y el manejo correcto de los síntomas, la reanimación volémica ≤ 30 mL/kg y la no preparación del colon, además de la adopción de procedimientos mínimamente invasivos y la promoción de la movilización temprana. La aplicación pionera del Proyecto ACERTO dio como resultado la reducción del tiempo de hospitalización, del uso de hemoderivados y de los casos de infección en el sitio quirúrgico, complicaciones operatorias y muertes^(5)^.

La comprensión profunda de los factores de riesgo de complicaciones es esencial para un enfoque más preciso y eficaz en el manejo posoperatorio de los pacientes sometidos a cirugías oncológicas, con el objetivo de mejorar continuamente los resultados clínicos y promover la calidad de vida. El objetivo de este estudio fue describir las complicaciones posoperatorias en pacientes sometidos a cirugías oncológicas, analizar el cumplimiento de las recomendaciones del proyecto ACERTO en estos pacientes y los factores de riesgo de muerte.

## MÉTODO

### Diseño del Estudio

Estudio de cohorte retrospectivo.

### Lugar del Estudio

Unidad de terapia intensiva del Hospital del Cáncer de Londrina, situado en la región norte de Paraná. Durante el periodo del estudio no se había implantado formalmente el proyecto ACERTO como protocolo de la institución de investigación. Las recomendaciones de este proyecto se utilizaron como referencia para la evaluación de la práctica clínica. En relación con la prevención de infecciones, la institución de investigación cuenta con un protocolo institucional formalmente implementado, que abarca diversas medidas preventivas alineadas con las recomendaciones del proyecto ACERTO, como la restricción del uso de drenajes y sondas, la movilización temprana, el apoyo nutricional temprano en el posoperatorio y la realización de auditorías para evaluar los resultados. El equipo del servicio de control de infecciones hospitalarias realiza auditorías diarias, supervisando la inserción y el mantenimiento de dispositivos invasivos, curativos, signos de infección respiratoria y el cumplimiento de las normas de higiene de las manos.

### Población y Criterios de Selección

Pacientes adultos de 18 años o más, sometidos a procedimientos quirúrgicos oncológicos e ingresados en la UTI en el postoperatorio inmediato. Se excluyeron de este estudio los pacientes sometidos a cirugías paliativas, así como aquellos que permanecieron ingresados en la UTI por menos de 24 horas. También se excluyó a los participantes con datos faltantes en el expediente médico que impedían el cálculo de las puntuaciones Escore fisiológico agudo simplificado (Simplified Acute Physiology Score 3 – SAPS 3) y Evaluación secuencial del fallo orgánica (Sequential Organ Failure Assessment – SOFA), además de la falta de información sobre la ocurrencia o no de complicaciones en el período perioperatorio.

### Definición de la Muestra

Muestra de conveniencia de los pacientes ingresados en el centro de estudio en el periodo comprendido entre julio y diciembre de 2021.

### Recolección de Datos

La información recogida abarcó los periodos preoperatorio, intraoperatorio y posoperatorio, extendiéndose hasta el resultado hospitalario. La categorización de las cirugías se realizó con base en la Tabla del Sistema de Gestión de Procedimientos, Medicamentos, Órtesis, Prótesis y Materiales Especiales (SIGTAP) del Sistema Único de Salud (SUS), una herramienta que permite acceder a la tabla de procedimientos del SUS.

En lo que respecta a las variables sociodemográficas, clínicas y epidemiológicas, se analizaron datos como la edad, el género, las condiciones preexistentes (tabaquismo, alcoholismo, consumo de drogas ilícitas, tratamiento oncológico y comorbilidades según la lista del Índice de Comorbilidad de Charlson *Comorbidity Index* ^(6)^.

En el preoperatorio, las variables incluyeron tiempo de ayuno, tiempo de hospitalización preoperatoria, necesidad de transfusión sanguínea y preparación del colon. Se consideraron tratamientos oncológicos la quimioterapia y la radioterapia realizadas en cualquier momento antes de la cirugía para el tratamiento del tumor primario.

En el procedimiento quirúrgico, se analizaron la clasificación del potencial de contaminación, el uso de ventilación mecánica (VM), el procedimiento quirúrgico específico, el tipo de anestesia, la administración de medicamentos, la reposición volémica y el uso de fármacos vasoactivos.

En el periodo posoperatorio se monitorizaron la necesidad de oxígeno, los datos de ventilación mecánica, el uso de agentes vasoactivos, la reposición volémica, el balance hídrico, la necesidad de hemodiálisis, la aparición de hemorragias, así como la administración de analgesia y antieméticos.

Las variables posoperatorias se registraron en cinco momentos distintos: posoperatorio inmediato (POI), primer posoperatorio (1.º PO), segundo posoperatorio (2.º PO), tercer posoperatorio (3.º PO) y último día de hospitalización en la UTI. Además de las variables mencionadas anteriormente, se investigó información pertinente, como el tiempo de estancia hospitalaria y en UTI, la aparición de complicaciones posoperatorias (infecciosas, cardiovasculares, respiratorias, gastrointestinales, renales, neurológicas, trastornos de la coagulación y electrolíticos), el inicio de la realimentación, la reintervención y el desenlace clínico. El resultado clínico se definió como el estado vital al alta hospitalaria, dividiéndose los grupos en supervivientes (alta) y no supervivientes (muerte).

El escore SAPS 3 evalúa la gravedad del estado de un paciente al ingresar en UTI para predecir la mortalidad hospitalaria. La puntuación SOFA evalúa el grado de disfunción orgánica en seis sistemas orgánicos principales (neurológico, respiratorio, cardiovascular, renal, hepático y hematológico). Estas puntuaciones fueron las variables elegidas por ser de uso habitual en la práctica clínica actual. La evaluación de los escores SAPS 3 se realizó en la primera hora de ingreso en la UTI, mientras que la SOFA se evaluó en los siguientes momentos: postoperatorio inmediato, 1.º postoperatorio, 2.º postoperatorio, 3.º postoperatorio y último día de ingreso en la UTI.

### Análisis y Tratamiento de los Datos

La normalidad de la distribución de las variables se verificó mediante la prueba de Shapiro-Wilk. Los resultados de las variables continuas se presentaron como media, desvío estándar (DE) o mediana e intervalo intercuartílico (IIC), dependiendo de la distribución de los datos.

Se utilizó la prueba t de Student para comparar las medias de las variables continuas con distribución normal y homogeneidad de varianzas. Para los datos con distribución no normal y/o heterogeneidad de varianzas, se aplicó la prueba no paramétrica (prueba U de Mann-Whitney).

Las variables categóricas se analizaron utilizando la prueba de chi-cuadrado y se presentaron como frecuencia absoluta y relativa. El nivel de significación adoptado fue del 5%. La evaluación de la asociación entre los posibles factores de riesgo (variables independientes) y la variable dependiente (resultado hospitalario) se realizó mediante la presentación de la Odds Ratio (OR) no ajustada y el intervalo de confianza del 95% (IC 95%). Estos datos se obtuvieron utilizando el modelo de regresión logística en modo Enter, que se caracteriza por el análisis bivariado. Posteriormente, se adoptó el modelo de regresión logística en el análisis multivariado. Para el análisis de regresión logística, la variable dependiente fue el desenlace no superviviente, las variables independientes fueron la edad, el sexo, las comorbilidades, las complicaciones, el SAPS 3, el SOFA en el POI y el tipo de cirugía.

El análisis de sensibilidad y especificidad de la puntuación SOFA para la discriminación del resultado se realizó mediante la curva ROC. Los resultados se presentan como área bajo la curva (AUC) e intervalo de confianza del 95% (IC 95%). Para comprobar las diferencias estadísticamente relevantes entre las curvas, se calcularon las diferencias de sus áreas dos a dos mediante el método de DeLong. Todo el proceso analítico se llevó a cabo utilizando el programa MedCalc® Statistical Software versión 22.018^(7)^.

### Aspectos Éticos

El presente estudio fue sometido y aprobado por el Comité de Ética en Investigación con Seres Humanos de la UEL (CEP-UEL) con el dictamen n.º 3.900.546; CAAE n.º: 28310420.6.0000.5231. Esta investigación obtuvo una exención del Término de Consentimiento Libre e Informado, justificada por el diseño y los objetivos del estudio.

## RESULTADOS

Para evaluar los factores de riesgo relacionados con las complicaciones en pacientes sometidos a cirugía oncológica, se seleccionó una muestra compuesta por pacientes ingresados consecutivamente en el centro del estudio entre julio y diciembre de 2021. Al aplicar los criterios de inclusión, la muestra resultó en 299 pacientes y, de estos, 70 casos fueron excluidos por los criterios del estudio, quedando 229 casos para su análisis. La exclusión se produjo en situaciones en las que los pacientes permanecieron en la UTI menos de 24 horas, se sometieron a cirugías paliativas o presentaban datos faltantes en los expedientes médicos.

La mediana de edad fue mayor entre los pacientes que no sobrevivieron (70 años, I 62–76) en comparación con los sobrevivientes (62 años, IIC 53–71; p = 0,025). Se observó una mayor frecuencia de sexo masculino (117; 51,0%) y 198 (86,4%) de los casos presentaban comorbilidades, destacando la hipertensión arterial sistémica (31; 57,2%). En cuanto a los hábitos, destaca la presencia de tabaquismo en 91 (39,7%) y alcoholismo en 27 (11,8%). Se observó que 59 (25,8%) pacientes se sometieron a algún tratamiento oncológico (radioterapia o quimioterapia) antes de la cirugía. El diagnóstico predominante asociado al procedimiento quirúrgico fue neoplasia maligna en el encéfalo, con un total de 38 (16,6%). A continuación, se observó neoplasia maligna en la unión rectosigmoidea, recto, ano y canal anal, con 24 (10,5%), y neoplasia maligna en la tráquea, bronquios y pulmones, con 18 (7,9%). La cirugía más frecuente fue la neurocirugía (61; 26,6%) (Tabla 1).

**Tabla 1:** Caracterización de los pacientes sometidos a procedimientos quirúrgicos ingresados en la unidad de cuidados intensivos – Londrina, PR, Brasil, 2022.

Variables	Frecuencia	%
Sexo Masculino	117	51,0
Presencia de comorbilidades		
HSA	31	57,2
DM	64	27,9
Cardiopatía	44	19,2
Hipotiroidismo	21	9,2
ERC	19	8,3
EPOC	16	7,0
EVP	2	0,9
ACV	2	0,9
Cirrosis hepática	2	0,9
VIH/sida	1	0,4
Tratamiento oncológico en cualquier momento antes de la cirugía	59	25,8
Hábitos		
Fumar	91	39,7
Alcoholismo	27	11,7
Prioridad de la cirugía		
Electiva	179	78,2
Urgencias	50	21,8
Potencial de contaminación		
Limpio	128	55,9
Potencialmente contaminado	77	33,6
Contaminado	23	10,0
Infectado	01	0,4
Tipo de anestesia		
Anestesia general	118	51,5
Anestesia combinada	100	43,7
Anestesia neuroaxial	11	4,8
Procedimiento quirúrgico Subgrupo Sigtap		
Neurocirugía	61	26,6
Aparato digestivo	38	16,6
Coloproctología	34	14,8
Cirugía torácica	24	10,5
Urología	23	10,0
Cabeza y cuello	18	7,9
Ginecología	10	4,4
Huesos y partes blandas	8	3,5
Mastología	4	1,7
Otras cirugías	4	1,7
Cirugía Oral y maxilofacial	3	1,3
Cirugía cutánea y plástica	1	0,4
Otras cirugías oncológicas	1	0,4

Siglas: HSA = Hipertensión Arterial Sistémica; DM = Diabetes Mellitus; EPOC = Enfermedad Pulmonar Obstructiva Crónica; ERC = Enfermedad Renal Crónica; EVP = Enfermedad Vascular Periférica; ACV = Accidente Cerebrovascular; VIH = Virus de Inmunodeficiencia Humana, Sigtap = (Sistema de Gestión de la Tabla de Procedimientos, Medicamentos, Órtesis/Prótesis y Materiales Especiales del SUS).

Los pacientes que presentaron complicaciones postoperatorias estuvieron expuestos a un tiempo quirúrgico mayor (205 min), mientras que en los pacientes que no presentaron complicaciones este tiempo fue menor (147 min) (p < 0,001). En el postoperatorio, el balance hídrico en los días del estudio fue: POI: mediana = 619,4 ml (IIC: -6,975–1.451,400); 1º PO: mediana = 925 mL (IIC: 0,425–1.745,200); 2º PO: mediana = 216 mL (IIC: -469,000–1.153,250); 3º PO: 528 mL (IIC: -444,600–1.193,750). El balance hídrico positivo en el 1o. PO fue mayor entre los no supervivientes, 1.478,4 ml (IIC: 969–218), en comparación con los supervivientes, 786,2 ml (IIC: -42–1.466; p = 0,010) (Figura 1).

**Figura 1:** Comparación del balance hídrico en los pacientes durante el posoperatorio en la unidad de cuidados intensivos.

Siglas: IPO = *postoperatorio inmediato*; 1PO = 1.º postoperatorio; 2PO = 2.º postoperatorio; 3PO = 3.º postoperatorio; BH = balance hídrico.

La frecuencia total de complicaciones postoperatorias fue de 157 (68,5%), siendo las más frecuentes las infecciosas, las respiratorias, las relacionadas con la coagulación y las renales. La mayoría de las complicaciones postoperatorias analizadas se asociaron a un aumento de la mortalidad, a excepción de las complicaciones gastrointestinales (Tabla 2). La tasa de mortalidad observada en esta muestra fue de 27 pacientes (11,8%).

**Tabla 2:** Análisis bivariante de las complicaciones postoperatorias como factor de riesgo de Muerte – Londrina, PR, Brasil, 2022.

Complicaciones	OR	IC 95%	p
Infecciosas	13,376	4,8124–37,1787	<0,001
Respiratorias	10,773	4,4536–26,0631	<0,001
Coagulación	8,000	1,0788–59,3265	0,041
Renal	6,018	2,5549–14,1776	<0,001
Quirúrgicas	4,935	2,1381–11,3946	0,002
Neurológicas	4,582	1,7476–12,0138	0,002
Electrolíticos	3,831	1,4872–9,8712	0,005
Cardiovasculares	2,702	1,1774–6,2033	0,019
Gástricas	1,987	0,8838–4,4680	0,096

Entre los 72 casos con complicaciones infecciosas, las más frecuentes fueron neumonía (34; 47,2%), infección del sitio quirúrgico (14; 19,4%), infección del torrente sanguíneo (12; 16,7%), infección urinaria (10; 13,9%) y foco de origen indeterminado (2; 2,8%). De los pacientes con complicaciones infecciosas, 14 (19,4%) se presentaron como sepsis y 30 (41,6%) como shock séptico. Entre los 229 pacientes del estudio, la proporción de pacientes que permanecieron en VM durante tres o más días fue de 20 (8,7%). De los 34 pacientes que desarrollaron neumonía como complicación infecciosa, 12 (35,3%) permanecieron en VM hasta el tercer día de seguimiento. De los 50 casos con complicaciones quirúrgicas, 34 (68,0%) precisaron transfusión sanguínea intraoperatoria, 13 (26,0%) tuvieron una fístula, 16 (32,0%) presentaron hemorragia postoperatoria y 7 (14,0%) rotura anastomótica. La variable duración de la estancia en la UCI fue de 44h19min entre los supervivientes y de 96h04min entre los no supervivientes. Se produjeron reingresos en la UCI en 16 (6,9%) casos y re-operaciones en 17 (7,4%). La puntuación SAPS 3 no difirió al comparar no supervivientes (mediana = 56; IIC: 44–70) y supervivientes (mediana = 54; IIC: 45–60; p = 0,129).

Se observó adherencia a las recomendaciones del proyecto ACERTO en relación con la reanimación precoz, la reposición de volumen y la profilaxis de náuseas y vómitos y su asociación con la aparición de complicaciones (Tabla 3). Hubo adherencia a la recomendación de reposición de volumen ≤ 30 mL/Kg intraoperatoria en 111 pacientes (56,6%) y postoperatoria en 206 pacientes (90,4%) y prescripción de profilaxis de náuseas y vómitos intraoperatoria en 213 pacientes (93,0%) y postoperatoria en 229 pacientes (100,0%). Hubo menos adherencia a la recomendación de abreviar el ayuno (73; 32,0%). Hubo una asociación entre el cumplimiento de las recomendaciones y la reducción de la frecuencia de complicaciones (Tabla 3).

**Tabla 3:** Análisis bivariado de los factores de riesgo de complicaciones postoperatorias en pacientes ingresados en la unidad de cuidados intensivos considerando las variables del proyecto Acelerando la Recuperación Postoperatoria Total – Londrina, PR, Brasil, 2022.

Variables	Total	Sin complicaciones	Con complicaciones	p
Retroalimentación postoperatoria				
Inmediata hasta 6 horas	73 (32,0%)	35 (47,9%)	38 (52,1%)	0,001
De 6 a 12 horas	37 (16,2%)	12 (32,4%)	25 (67,6%)	
De 12 a 24 horas	58 (25,4%)	16 (27,6%)	42 (72,4%)	
De 24 a 48 horas	38 (16,7%)	6 (15,8%)	32 (84,2%)	
Más de 48 horas	21 (9,2%)	2 (9,5%)	19 (90,5%)	
No se produjo	1 (0,4%)	(–)	1 (100,0%)	
Reposición de volumen intraoperatorio				
≤ 30 mL/Kg	111(56,6%)	41 (36,9%)	70 (63,1%)	0,005
> 30 mL/Kg	85 (43,4%)	16 (18,8%)	69 (81,2%)	
Reposición de volumen en el postoperatorio inmediato				
≤ 30 mL/Kg	206 (90,4%)	69 (33,5%)	137 (66,5%)	0,057
> 30 mL/Kg	22 (9,6%)	3 (13,6%)	3 (13,6%)	
Reposición de volumen				
No	34 (14,8%)	16 (47,1%)	18 (52,9%)	0,033
Sí	195 (85,2%)	56 (28,7%)	139 (71,3%)	
Saldo positivo en el balance hídrico postoperatorio inmediato				
No	201 (87,8%)	69 (34,3%)	132 (65,7%)	0,011
Sí	28 (12,2%)	3 (10,7%)	25 (89,3%)	
Profilaxis de náuseas y vómitos intraoperatorios				
No	16 (7,0%)	2 (12,5%)	14 (87,5%)	0,091
Sí	213 (93,0%)	70 (32,9%)	143 (67,1%)	
Profilaxis postoperatoria de náuseas y vómitos				
No	(–)	(–)	(–)	<0,001
Sí	229 (100,0%)	72 (31,4%)	157 (68,6%)	

En el análisis multivariante, los factores de riesgo independientes de muerte fueron: edad (OR = 1,0388; IC 95%: 1,0034–1,0754; p = 0,031) y SOFA en el postoperatorio inmediato (OR = 1,2136; IC 95%: 1,0620–1,3867; p = 0,004) (Tabla 4).

**Tabla 4:** Análisis bivariado y multivariado para el resultado Muerte – Londrina, PR, Brasil, 2022.

	Bivariado			Multivariado		
Variables	OR	IC 95%	p	OR	IC 95%	p
Edad	1,037	1,0049–1,0710	0,023	1,038	1,0034–1,0754	0,031
Sexo	1,035	0,4634–2,3123	0,933			
Comorbilidades	4,534	0,5928–34,6894	0,145			
Complicaciones	30,55	1,8366–508,3583	0,017			
SAPS 3	1,035	1,0058–1,0668	0,019			
SOFA en POI	1,217	1,0687–1,3876	0,003	1,213	1,0620–1,3867	0,004
Cirugía	1,604	0,6566–3,9186	0,299			

El análisis de la curva ROC mostró un AUC para el SOFA en el postoperatorio inmediato de 0,625, en el 1º PO de 0,629, en el 2º PO de 0,687 y en el 3º PO de 0,721. Las puntuaciones SOFA en el 2º PO (0,013) y en el 3º PO (p = 0,002) mostraron un mayor poder discriminatorio para predecir la mortalidad hospitalaria en comparación con las demás (Figura 2).

**Figura 2:** Curva ROC para evaluar la eficacia de la puntuación SOFA como predictor de mortalidad postoperatoria durante la hospitalización en la UCI.

Siglas: SOFA = *Sequential Organ Failure Assessment (Evaluación secuencial de fallo orgánico)*; UTI = Unidad de Cuidados Intensivos; IPO = postoperatorio inmediato; 1PO = 1.º postoperatorio; 2PO = 2.º postoperatorio; 3PO = 3.º postoperatorio).

## DISCUSIÓN

En este estudio, el cumplimiento de las recomendaciones del protocolo ACERTO se asoció con una reducción de las complicaciones postoperatorias en los pacientes oncológicos ingresados en la Unidad de Terapia Intensiva. Se observó una alta frecuencia de complicaciones, que se asociaron a un mayor riesgo de muerte.

El estudio de Silva Jr et al.^(8)^ observó que la mayoría de los pacientes tenían una edad media de 62 años y eran en su mayoría hombres, al igual que los resultados encontrados en el presente estudio. En la muestra analizada, el 86,5% de los pacientes presentaban comorbilidades. Otros autores también muestran que la edad avanzada representa un factor predictivo de muerte cuando se asocia a múltiples comorbilidades, principalmente en lo que se refiere a la diabetes mellitus, la hipertensión arterial y el uso de polifarmacia, que contribuyen al desarrollo de lesiones renales agudas y, en consecuencia, a una mayor probabilidad de muerte entre los pacientes de cuidados intensivos^(9,10)^.

Se observó una alta proporción de adhesión a las recomendaciones del proyecto ACERTO para la reposición volémica ≤ 30 mL/kg en las fases iniciales de la reanimación hemodinámica y la profilaxis de las náuseas y los vómitos. Aunque el lugar del estudio no cuenta con un protocolo institucional formal, estas prácticas recomendadas se han incorporado a la rutina clínica. En una publicación reciente se sugirió dividir la reanimación hemodinámica en cuatro fases, recomendándose en las dos fases iniciales la reposición de fluidos con el objetivo de una rápida expansión del volumen intravascular para mejorar la perfusión tisular^(11)^. Esta expansión inicial puede dar lugar a un balance hídrico positivo en estas fases iniciales. En las fases siguientes de estabilización y desescalada, el objetivo es un balance hídrico cero o incluso negativo, con el fin de prevenir los efectos adversos de la sobrecarga de volumen. En los pacientes del presente estudio, podemos observar un balance hídrico positivo en los primeros días del postoperatorio, compatible con esta recomendación, pero se esperaría un valor cero o negativo en el 3^er^ día del postoperatorio. No hubo pacientes con prescripción de reanimación volémica en los pacientes del presente estudio en ese día del 3.^er^ PO, por lo que lo que explicaría este balance hídrico positivo serían otros fluidos utilizados en el tratamiento del paciente grave, como fluidos para la dilución de medicamentos, entre otros. Souza y colaboradores demostraron que los fluidos que no se utilizan para la reanimación hemodinámica pueden ser los principales contribuyentes a un balance hídrico positivo durante la reanimación hemodinámica^(12)^.

Los pacientes quirúrgicos candidatos a procedimientos digestivos, principalmente en oncología, presentan una alta prevalencia de desnutrición aislada o asociada a sarcopenia^(13)^. En el presente estudio, se observó una menor adherencia a la realimentación temprana en las primeras 6 horas. También se observó un efecto dosis-respuesta, es decir, cuanto más prolongado era el ayuno en el período posoperatorio, mayor era la frecuencia de las complicaciones observadas. El ayuno prolongado pre y posoperatorio tradicionalmente impuesto por la cirugía puede agravar la respuesta orgánica y el estado nutricional, predisponiendo al paciente a una mayor respuesta al trauma y al deterioro de la respuesta inmunológica. El protocolo ACERTO recomienda, con un alto grado de recomendación y un alto nivel de evidencia, que la realimentación oral o enteral después de una operación abdominal electiva debe ser temprana (dentro de las 24 horas posteriores a la operación), siempre que el paciente esté hemodinámicamente estable. En operaciones como la videocolecistectomía, las hernioplastias y las cirugías ano-orificiales, se recomienda el inicio inmediato de la dieta y la hidratación oral, sin necesidad de hidratación intravenosa^(14)^.

En el presente estudio, la reposición volémica de cristaloides ≤ 30 mL/kg en las primeras fases de la reanimación hemodinámica se asoció con una menor incidencia de complicaciones, lo que corrobora los hallazgos de otros autores^(12)^. La sobrecarga de cristaloides puede causar edema generalizado de los tejidos, lo que conlleva varias consecuencias clínicas, como la alteración de la función cardiopulmonar, la disminución de la oxigenación sanguínea y efectos negativos en todo el organismo. En el tracto digestivo, esto da lugar a edema esplácnico, aumento de la presión intraabdominal, disminución del flujo mesentérico, mantenimiento del íleo paralítico, aumento de la permeabilidad de la mucosa y compromiso del proceso de cicatrización^(15)^.

Otro hallazgo del presente estudio fue la alta adherencia a las recomendaciones de profilaxis para las náuseas y los vómitos durante el intraoperatorio y el postoperatorio inmediato. Se observó una mayor incidencia de complicaciones entre los casos que no recibieron profilaxis. Un estudio que analizó los factores de riesgo de náuseas y vómitos posoperatorios (NVPO) constató que la incidencia de NVPO es alta, alcanzando el 37,5% después de la anestesia general^(16)^. Sin embargo, esta incidencia puede llegar al 70% en pacientes de alto riesgo^(17)^. Cabe destacar que la aparición de náuseas y vómitos retrasa la realimentación del paciente, merma su confianza y aumenta la necesidad de líquidos intravenosos y el riesgo de dehiscencia de la herida quirúrgica, lo que puede retrasar el alta hospitalaria^(15)^.

Se identificó una mortalidad del 11,8% durante el periodo de hospitalización, similar a la del estudio realizado por Silva Jr et al.^(8)^, que mostró una tasa de mortalidad en la UTI del 4,9%, una tasa de mortalidad hospitalaria del 8,9% y una tasa de mortalidad a los 28 días de seguimiento del 9,6%.

Las complicaciones posoperatorias asociadas a mayores riesgos de muerte fueron: infecciosas, respiratorias, relacionadas con la coagulación y renales. Entre las complicaciones posoperatorias, las renales son responsables de una proporción considerable de la morbilidad y mortalidad relacionadas con las cirugías^(18,19)^. Un estudio de cohorte prospectivo y multicéntrico constató la incidencia total de complicaciones posoperatorias, con una mayor ocurrencia de complicaciones cardiovasculares, renales, respiratorias y neurológicas, respectivamente^(8)^.

El análisis multivariante identificó factores de riesgo independientes para la muerte, como la edad y la puntuación SOFA. Cabe destacar la importancia de implementar estrategias para reducir la mortalidad, mediante la evaluación continua de estas puntuaciones por parte del personal asistencial y directivo de las UTI, a tiempo para tratar o reducir las disfunciones orgánicas. En un estudio prospectivo que analizó a 100 pacientes con sepsis ingresados en la UTI con evidencia de disfunción orgánica, las puntuaciones iniciales más altas de SAPS 2 y SOFA se asociaron con peores resultados^(20)^.

Como limitación del estudio, cabe destacar que se trata de un estudio retrospectivo y de un solo centro, lo que puede ser restrictivo y dar lugar a datos incompletos sobre variables de interés, como las relativas a las recomendaciones de ACERTO. El hecho de que se haya realizado en un solo centro limita el número de participantes y reduce la validez externa de los resultados. En cuanto al análisis específico del manejo de fluidos, no fue posible incluir datos del BH intraoperatorio, ya que se encontraron registros de la prescripción de fluidos en ese periodo, pero no había datos sobre las pérdidas, lo que imposibilitó este cálculo. Además, el tamaño limitado de la muestra puede haber dificultado la identificación de factores de riesgo relevantes para la muerte, aunque se haya probado con el modelo estadístico con mejor rendimiento. La fortaleza de esta investigación radica en el hecho de que existen pocos datos latinoamericanos, especialmente en el área oncológica.

Cabe destacar la implicación de estos hallazgos para la práctica clínica, lo que demuestra la importancia de implementar el proyecto ACERTO en el hospital del lugar de estudio, con el objetivo de reducir la morbilidad y la mortalidad.

## CONCLUSIÓN

En este estudio se identificó una alta frecuencia de complicaciones en el periodo posoperatorio y una tasa de mortalidad hospitalaria similar a la de otros autores. Las complicaciones asociadas a un mayor riesgo de muerte fueron infecciosas, respiratorias, renales y relacionadas con la coagulación. El cumplimiento de las recomendaciones del protocolo ACERTO se asoció con una reducción de las complicaciones posoperatorias en los pacientes oncológicos. Se observaron como factores de riesgo independientes para la muerte la edad y la puntuación SOFA.

## DISPONIBILIDAD DE DATOS

Los datos de la presente investigación se han depositado en el link: https://doi.org/10.48331/SCIELODATA.D4NF6L.
